# Role of lactylation and immune infiltration in atherosclerosis: novel insights from bioinformatics analyses

**DOI:** 10.3389/fgene.2025.1520325

**Published:** 2025-04-03

**Authors:** Jintao Qian, Qing Zhou

**Affiliations:** Department of Cardiothoracic surgery, Nanjing Drum Tower Hospital, Affiliated Hospital of Medical School, Nanjing University, Nanjing, China

**Keywords:** atherosclerosis, lactylation, immune infiltration, risk prediction, biomarkers

## Abstract

**Introduction:**

The existing evidence indicates that atherosclerosis (AS) plays a pivotal role in the progression and exacerbation of cardiovascular diseases and their associated complications. Current diagnostic and therapeutic strategies for atherosclerosis are limited in their ability to facilitate early detection and personalized treatment. This study employs a systems biology approach to investigate the role of lactylation-related genes (LRGs) in the pathogenesis of atherosclerosis, while considering the well-established correlation between inflammatory responses and atherosclerosis development.

**Methods:**

In this study, we utilized datasets obtained from the Gene Expression Omnibus (GEO) as well as data from previous studies on lactylation-related genes (LRGs). Following this, we identified 17 lactylation related genes associate with atherosclerosis (AS-LRGs) from the GSE100927 dataset. Subsequently, we employed the validation dataset (GSE43292) to assess these 17 AS-LRGs, resulting in the identification of 12 more reliable candidate genes. These genes were further analyzed for functional enrichment through Gene Ontology (GO) annotation, Kyoto Encyclopedia of Genes and Genomes (KEGG) pathway analysis, and gene set enrichment analysis (GSEA). To elucidate the potential utility of AS-LRGs in diagnosing high-risk plaques, we assessed their expression in both early and late stages of atherosclerosis, as well as in high- and low-risk plaques. We then constructed interaction networks to elucidate the potential regulatory relationships among LRGs, miRNAs, transcription factors, and drugs. Finally, we utilized the single sample Gene Set Enrichment Analysis (ssGSEA) method to investigate immune infiltration in AS and evaluate the levels of immune cell infiltration.

**Results:**

We identified 12 lactylation-related genes that are more reliably associated with atherosclerosis: five upregulated genes (LSP1, IKZF1, MNDA, RCC2, and WAS) and seven downregulated genes (CSRP2, PPP1CB, CSRP1, HEXIM1, CALD1, PDLIM1, and RANBP2).

**Discussion:**

This study elucidates the pivotal role of lactylation in atherosclerosis (AS) and establishes a robust foundation for future research into targeted therapies and clinical applications of the identified biomarkers.

## 1 Introduction

Atherosclerosis is a progressive condition characterized by diverse structural modifications in the walls of large and medium-sized arteries, ultimately resulting in the development of atherosclerotic plaques (Camaré et al., 2017). It poses a significant threat to both individual patients and public health, as it is one of the primary causes of cardiovascular diseases. As demonstrated by prior studies, atherosclerotic cardiovascular disease constitutes a significant global contributor to mortality (Barquera et al., 2015). Furthermore, atherosclerotic plaques serve as the pathophysiological foundation for nearly all arterial vascular diseases (Pi et al., 2024). Current diagnostic and therapeutic approaches for atherosclerosis include pharmacological treatments, lifestyle modifications, and surgical interventions; however, significant limitations remain, such as inaccurate diagnoses and suboptimal therapeutic outcomes, which necessitate further attention (Libby et al., 2019; Shamaki et al., 2022).

Lactylation, which is regulated by lactate concentrations, has recently been recognized as a novel component of the epigenetic landscape. This finding not only facilitates extensive research into lactate metabolism but also offers essential reference points for subsequent functional and mechanistic studies (Liu et al., 2022). Prior research has demonstrated that lactylation modification is crucial in the pathogenesis of various diseases, including cancer (Li F. et al., 2024) and cardiovascular disorders (Wang N. et al., 2022). Integrating the distribution and metabolic characteristics of cells within atherosclerotic plaques, lactylation modification may present a novel strategy for targeted interventions in atherosclerosis (Xu et al., 2023). However, the role of lactylation modification in atherosclerosis remains a topic of active debate within the scientific community, with varying perspectives on its significance and mechanisms. Recent studies have demonstrated that lactic acid-mediated lactylation modification promotes the development of atherosclerosis (Dong et al., 2024); however, other research suggests that under specific conditions, lactylation modification may exert an inhibitory effect on atherosclerotic progression (Zhang et al., 2024).

In this study, we systematically compiled published data related to lactylation and conducted an in-depth analysis of gene expression levels across multiple atherosclerotic databases to identify potential diagnostic markers and therapeutic targets. Additionally, we elucidated a range of immune cells associated with atherosclerosis through bioinformatics analysis, highlighting their significant roles in disease progression and their interactions with immune responses. In conclusion, the intersection of lactylation and atherosclerosis represents a promising frontier for exploration in contemporary biomedical research. By focusing on the expression and functional implications of lactylation-related genes (LRGs) in atherosclerosis (AS), this study aims to address existing knowledge gaps and lay the foundation for future investigations into targeted therapeutic strategies.

## 2 Materials and methods

### 2.1 Identification of differentially expressed genes between atherosclerotic and healthy arteries

A total of four AS-related gene expression matrices (GSE100927, GSE43292, GSE28829, and GSE163154) were obtained from the GEO database (https://www.ncbi.nlm.nih.gov/geo/). In the GSE100927 dataset (Steenman et al., 2018), atherosclerotic plaques and control arteries without such lesions (obtained from deceased organ donors) were collected from the carotid, femoral, and infra-popliteal arteries. Total RNA was extracted from the samples using standard protocols and subsequently hybridized to microarrays for further analysis. The “limma (Ritchie et al., 2015)” R package was employed to normalize the data and identify differentially expressed genes (DEGs). The significance criteria were defined as −0.58 < log2 fold change (FC) < 0.58, accompanied by a p-value of less than 0.05. In R version 4.2.1, the “ggplot2” and “ComplexHeatmap (Gu et al., 2016)” packages were utilized to generate volcano plots and heatmaps for the differentially expressed genes (DEGs). A list of 332 lactylation-related genes (LRGs) was obtained from previously published reports (Cheng et al., 2023). The collected LRG data were then integrated with the results of differential expression analysis of genes (DEGs) between the disease and control groups in the GSE100927 dataset, and these genes were classified as AS-related LRGs (AS-LRGs). The results were visualized utilizing the “ggplot2” and “VennDiagram” packages.

### 2.2 Analysis of LRGs expression in the validation dataset and receiver operating characteristic (ROC) curves

The GSE43292 dataset was utilized as the validation dataset. In GSE43292 dataset (Ayari and Bricca, 2013), a total of 34 patients who underwent carotid endarterectomy at the University Hospital of Lyon participated in this study. Carotid endarterectomy samples were obtained in the operating room and immediately divided into two fragments: the atheroma plaque and macroscopically intact tissue. We evaluated the expression levels of AS-LRGs in both affected and control groups, using the Wilcoxon rank-sum test for statistical analysis. The ROC analysis on the dataset was performed using the “pROC (Robin et al., 2011)” package. Statistical analysis of paired samples was conducted using a paired t-test. All visualizations were generated using the “ggplot2” package.

### 2.3 Functional and pathway enrichment analysis

Functional and pathway enrichment analyses were conducted using the Gene Ontology (2015) and Kyoto Encyclopedia of Genes and Genomes (KEGG) (Kanehisa et al., 2022) databases, employing the R package “clusterProfiler (Yu et al., 2012)” based on significant AS-LRGs (P < 0.05).

### 2.4 Gene set enrichment analysis (GSEA)

Gene sets redefined from the MSigDB database (https://www.gsea-msigdb.org/gsea/msigdb/collections.jsp) were utilized to analyze the distribution patterns of genes within a gene expression profile. This profile is ranked based on the correlation between gene expression and phenotypic characteristics, thereby evaluating the contributions and associations of these genes with the phenotype (Subramanian et al., 2005). We retrieved the gene sets “c2.cp.kegg.v2022.1.Hs.symbols” and “c5.all.v2022.1.Hs.symbols” from the MSigDB database and utilized the “clusterProfiler (Yu et al., 2012)” package in R to perform the analysis.

### 2.5 Construction of interaction networks involving AS-LRGs, associated microRNAs, transcription factors, and drugs

The TarBase v9.0 (Skoufos et al., 2024) and ENCODE (2012) databases were employed to identify the miRNAs and transcription factors associated with AS-LRGs, which were subsequently visualized using the NetworkAnalyst (2012) platform. We utilized the Drug Gene Interaction Database (DGIdb) (Cannon et al., 2024) (https://www.dgidb.org) to predict the relationships between genes and drugs, which were visualized using Cytoscape (Shannon et al., 2003).

### 2.6 Analysis of immune infiltration

The immune infiltration within the dataset was assessed using the single-sample GSEA (ssGSEA) algorithm from the R package GSVA (Hänzelmann et al., 2013), incorporating 24 immune cell markers detailed in an Immunity article (Bindea et al., 2013). We conducted the Wilcoxon rank-sum test to examine the differences between the normal group and the AS group. Spearman’s correlation analysis was conducted to assess the relationship between AS-LRGs and the level of immune infiltration in AS tissues. All visualizations were generated utilizing “ggplot2” package.

### 2.7 Analysis of AS-LRGs expression in the GSE28829 and GSE163154 datasets, with ROC curve evaluation

In the GSE28829 database, Seventeen samples of atherosclerotic tissue were collected from carotid artery segments, including nine samples representing early lesions and eight samples representing advanced lesions, sourced from the Maastricht Pathology Tissue Collection for inclusion in this study (Döring et al., 2012). The GSE28829 database was utilized to analyze the differential expression of AS-LRGs between early and advanced lesions. In GSE163154 database, they conducted an analysis of the genetic profiles of atherosclerotic lesion segments categorized as low-risk or high-risk in patients undergoing carotid endarterectomy, based on the presence or absence of intraplaque hemorrhage (Jin et al., 2021). The GSE163154 database was employed to analyze the differential expression of AS-LRGs in the absence and presence of intraplaque hemorrhage. The statistical and presentation methodologies utilized in this study are consistent with those previously described.

## 3 Results

### 3.1 Identification of lactylation-related genes in atherosclerosis

**FIGURE 1:**
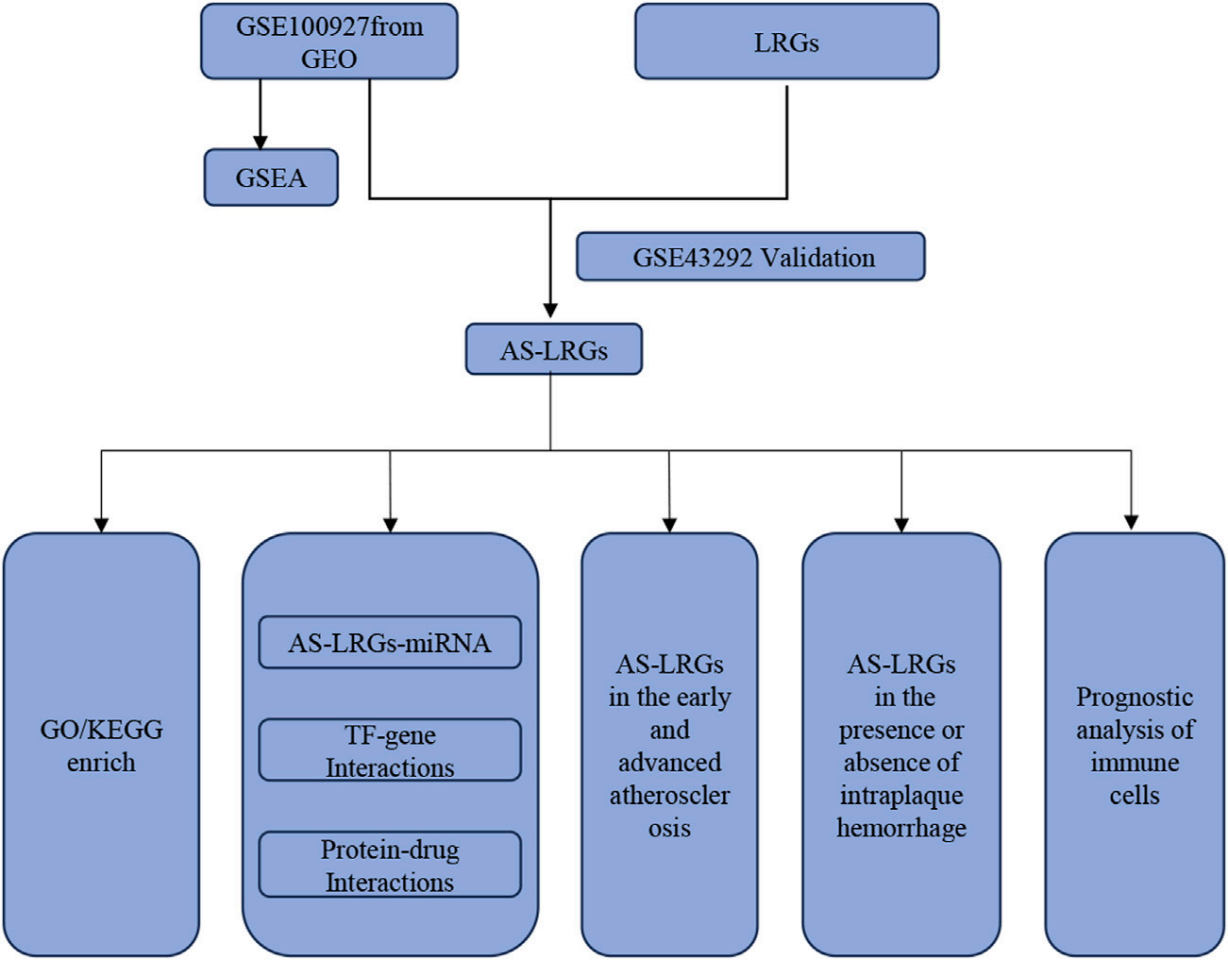
Schematic representation of the Study’s flow diagram.

**FIGURE 2:**
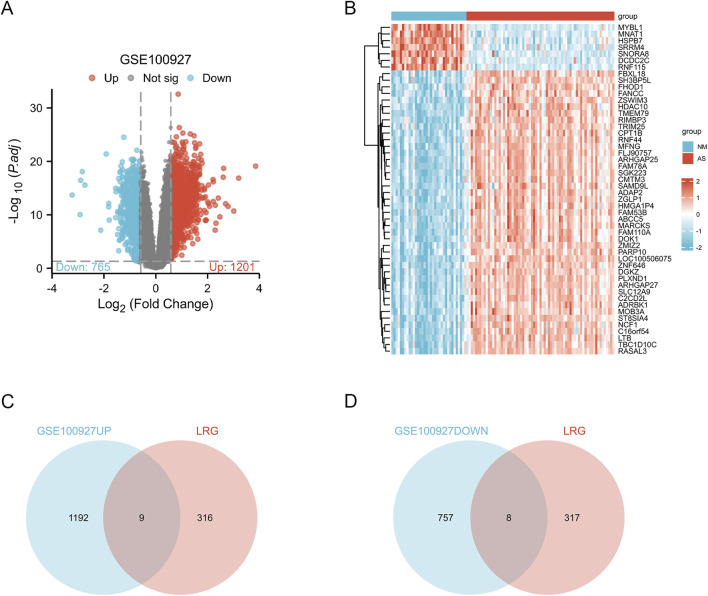
Analysis of Differential Gene Expression and lactylation-related genes (LRGs) Associated with Atherosclerosis **(A)**The volcano plot illustrates the differentially expressed genes (DEGs) between normal and atherosclerotic arteries within the GSE100927 dataset. In this representation, blue and red hues denote downregulated and upregulated genes, respectively, while grey nodes signify genes that do not exhibit significant differential expression (|log2 fold change (FC)| > 0.58 and p-value <0.05). **(B)** Heatmaps depict AS-related DEGs derived from the GSE100927 dataset. The vertical axis represents the specific DEGs, while the horizontal axis denotes patient identifiers. Blue signifies low gene expression levels, whereas red indicates high expression levels; blue bars correspond to normal arteries and red bars represent atherosclerotic arteries. **(C)** The intersection of upregulated genes in atherosclerotic arteries and lactylation-related genes revealed nine genes associated with both conditions: FABP5, LSP1, IKZF1, MNDA, CRABP2, ARID3A, RCC2, WAS, and HMGA1. **(D)** The intersection of downregulated genes in atherosclerotic arteries and lactylation-related genes identified eight genes associated with both conditions: CSRP2, PPP1CB, CSRP1, HIST1H1C, HEXIM1, CALD1, PDLIM1, and RANBP2.

**FIGURE 3:**
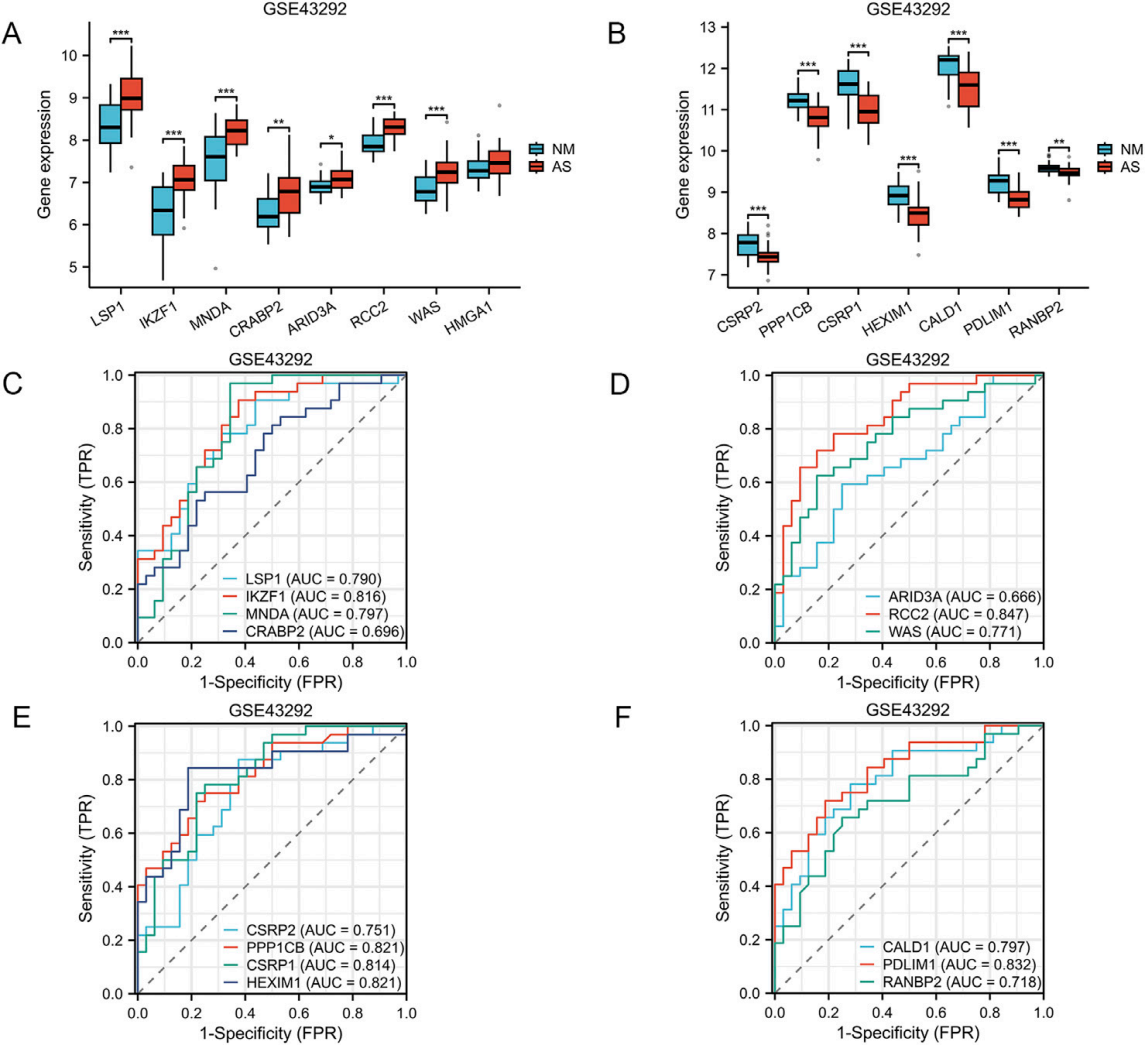
Verification of Expression Levels of Atherosclerosis-Associated Lactylation-Related Genes (AS-LRGs) in GSE43292 Dataset **(A, B)** The expression levels of the 8 upregulated AS-LRGs and 7 downregulated AS-LRGs in the GSE43292 dataset are illustrated, with blue boxes representing the normal group and red boxes denoting the disease group. **(C–F)** Receiver operating characteristic (ROC) curve analysis for 7 upregulated AS-LRGs and 7 downregulated AS-LRGs in the GSE43292 dataset . Based on the ROC analysis results, we identified 5 upregulated AS-LRGs and 7 downregulated AS-LRGs (AUC >0.7).

**FIGURE 4:**
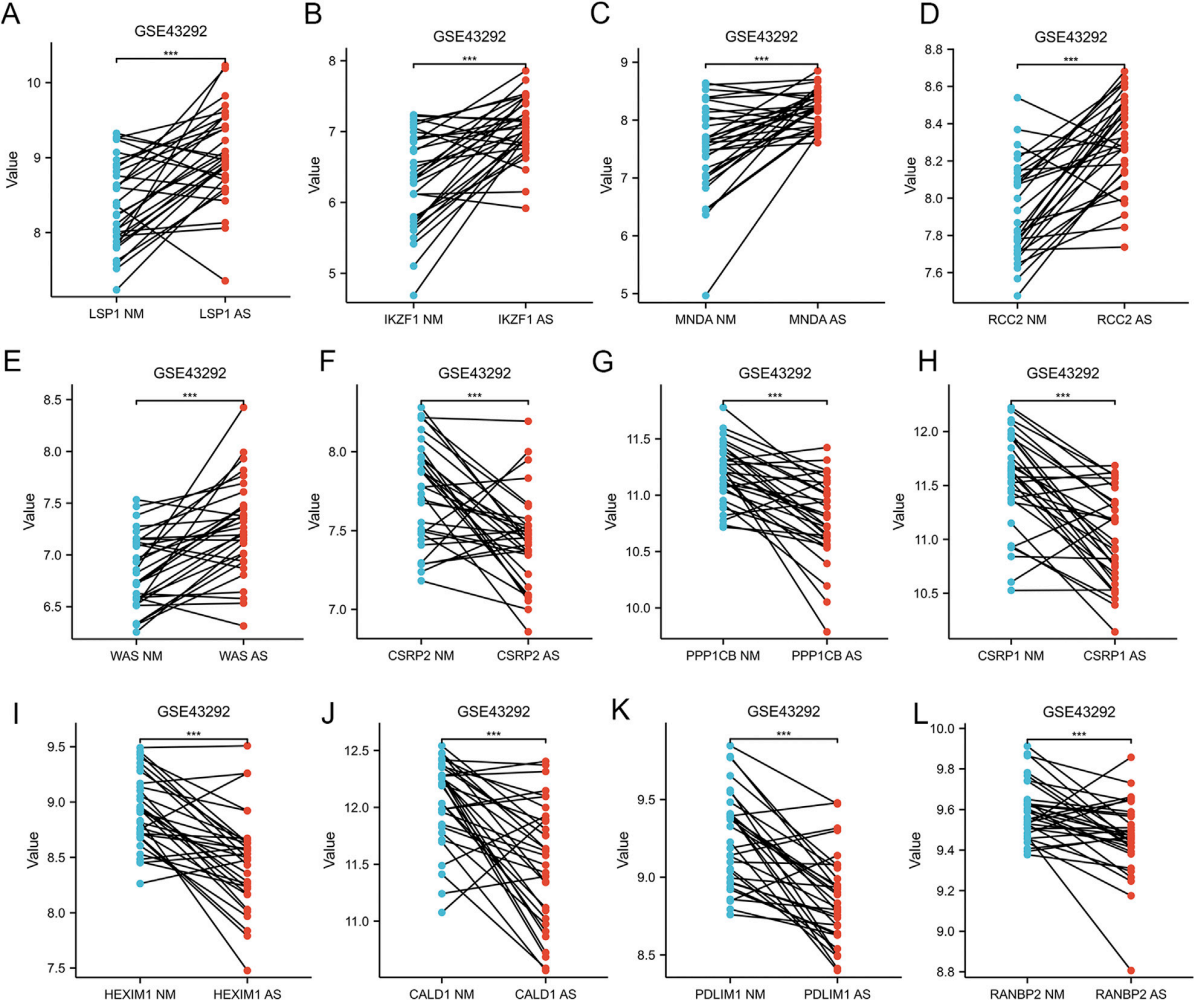
Paired Sample Analysis of Five Upregulated and Seven Downregulated AS-LRGs in the GSE43292 Dataset **(A–E)** Paired sample analysis of five upregulated AS-LRGs. **(F–L)** Paired sample analysis of seven downregulated AS-LRGs. The blue notes represent the normal group, while the red notes denote the disease group.

Figure 1 provides a comprehensive overview of the workflow. The differentially expressed genes (DEGs) in the GSE100927 dataset were visualized using volcano plots (Figure 2A). As observed, there were 1,201 upregulated DEGs and 765 downregulated DEGs. A classification heatmap was created to clearly present these results (Figure 2B). Additionally, by integrating the LRGs with the upregulated and downregulated DEGs from the GSE100927 dataset, we identified 9 upregulated genes (FABP5, LSP1, IKZF1, MNDA, CRABP2, ARID3A, RCC2, WAS, and HMGA1) and 8 downregulated genes (CSRP2, PPP1CB, CSRP1, HIST1H1C, HEXIM1, CALD1, PDLIM1, and RANBP2) that are common to both DEGs and LRGs and are associated with atherosclerosis (Figures 2C, D). Subsequently, we assessed the expression levels of these genes in the GSE43292 dataset. Among the identified genes, FABP5 and HIST1H1C were not detected as independent samples in the GSE43292 dataset. Subsequently, the expression of the 8 upregulated genes and 7 downregulated genes in the GSE43292 dataset were illustrated using box plots (Figures 3A, B). In the validation dataset (GSE43292), the 5 upregulated genes identified as having diagnostic value were: LSP1, IKZF1, MNDA, RCC2, and WAS (AUC >0.7) (Figures 3C, D); the 7 downregulated genes identified as having diagnostic value were: CSRP2, PPP1CB, CSRP1, HEXIM1, CALD1, PDLIM1 and RANBP2 (AUC >0.7) (Figures 3E, F). Subsequently, we conducted paired-sample t-tests on the 5 upregulated genes and 7 downregulated genes within the validation dataset, resulting in significant differences for all genes (Figures 4A–L).

### 3.2 Functional enrichment analysis of AS-LRGs and gene set enrichment analysis (GSEA)

**FIGURE 5:**
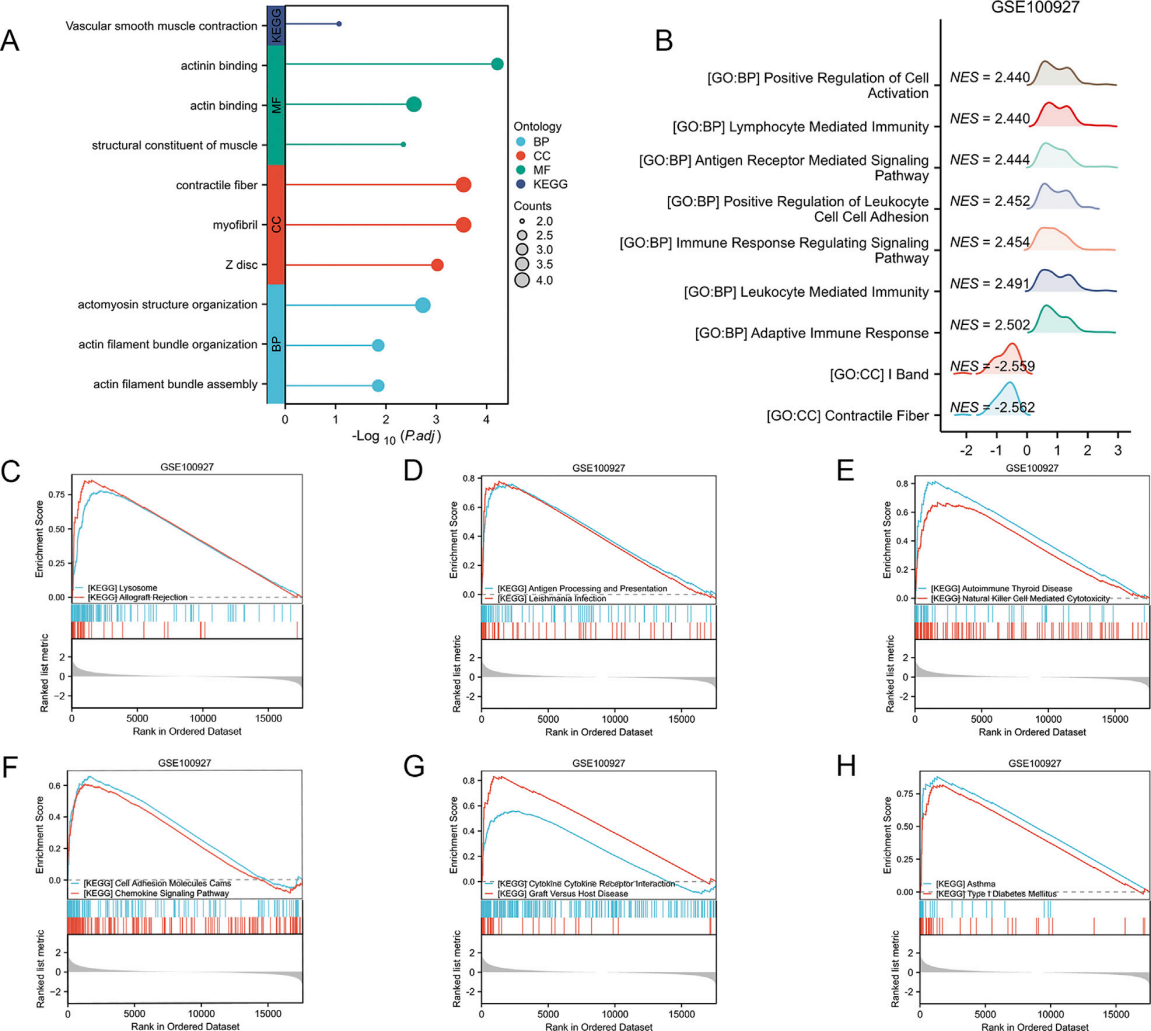
Functional Enrichment Analysis of AS-LRGs, along with GSEA-GO and GSEA-KEGG Analyses of the GSE100927 Dataset **(A)** Gene Ontology (GO) enrichment analysis and KEGG pathway enrichment analysis for 12 AS-LRGs. **(B)** GSEA–GO analysis of GSE100927 data. **(C–H)** GSEA–KEGG analysis of GSE100927 data.

The gene ontology (GO) analysis revealed significant enrichment in a multitude of biological processes, cellular components, and molecular functions. The KEGG analysis of the AS-LRGs revealed significant enrichment across a diverse array of pathways. The results of the GO and KEGG analyses for AS-LRGs are presented in Figure 5A. To evaluate the impact of gene expression levels on AS, Gene Set Enrichment Analysis (GSEA) was conducted using the GSE100927 dataset to elucidate the relationships between gene expression and related biological processes, cellular components, and molecular functions. The findings indicated that in the GSE100927 dataset, the genes were primarily associated with contractile fiber, I band, adaptive immune response, leukocyte-mediated immunity, immune response-regulating signaling pathways, and other biological functions (Figure 5B). The biological pathways predominantly regulated by genes in the GSE100927 dataset include those associated with lysosome, allograft rejection, antigen processing and presentation, leishmania infection, autoimmune thyroid disease, natural killer cell mediated cytotoxicity, cell adhesion molecules, chemokine signaling pathway, cytokine-cytokine receptor interaction, graft versus host disease, asthma, and type I diabetes mellitus (Figures 5C–H).

### 3.3 Network analysis of AS-LRGs and their associated miRNAs, transcription factors, and drugs

**FIGURE 6:**
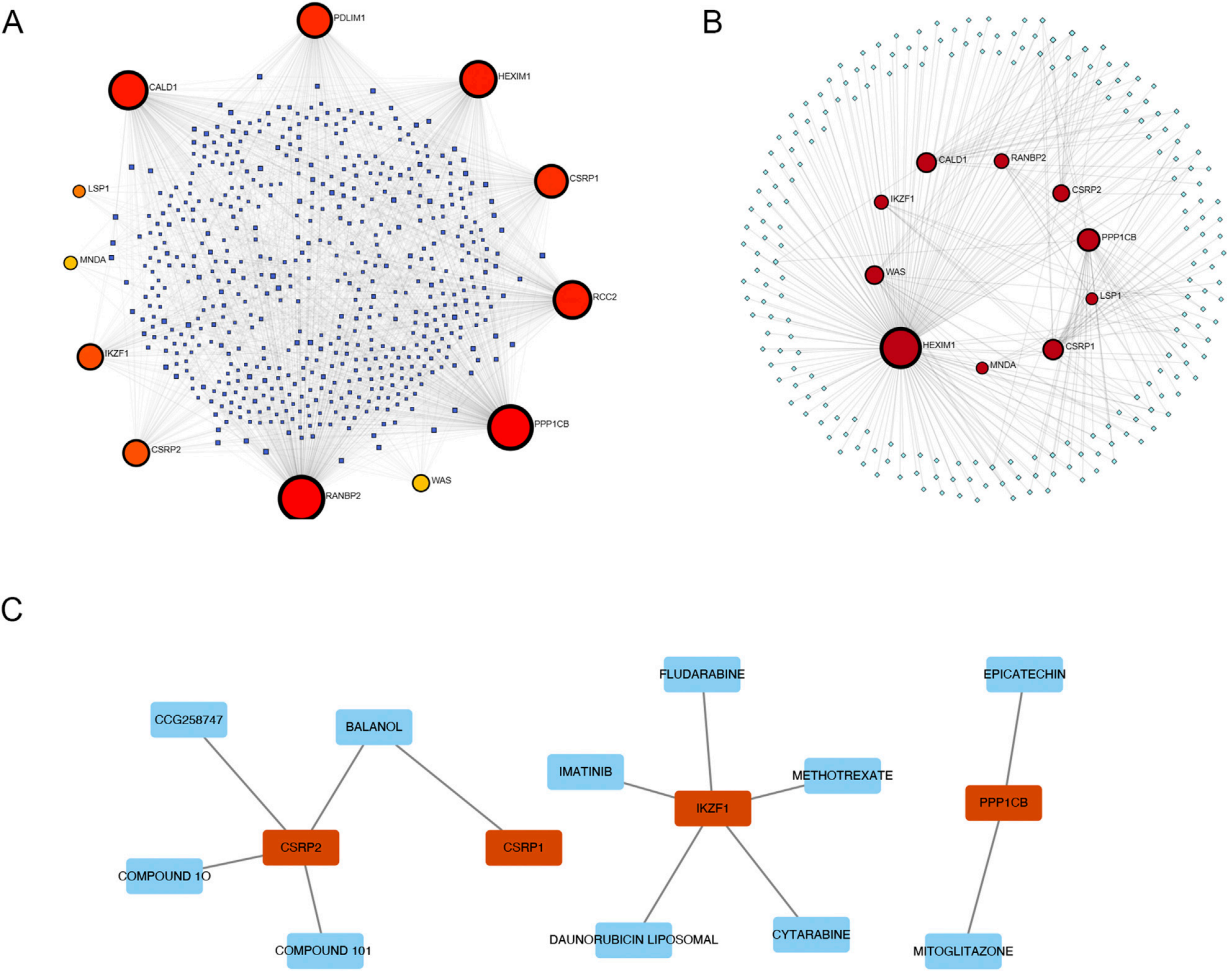
Network Visualization Illustrating the Interactions of AS-LRGs with miRNAs, Transcription factors (TFs), and Drugs **(A)** A network diagram illustrating the interactions between AS-LRGs and miRNAs, with blue nodes representing miRNAs and other nodes denoting AS-LRGs. **(B)** A network diagram for AF-PRGs and TFs, where green nodes represent TFs and other nodes denote AS-LRGs. **(C)** Relationship between AS-LRGs and drugs, The green nodes denote drugs, while the red nodes represent AS-LRGs.

We constructed an interaction network between AS-LRGs and miRNAs, encompassing 12 genes and 561 miRNAs (Figure 6A). The top five predictions of the AS-LRG-miRNA interaction network included: RANBP2 (targeted by 294 miRNAs), PPP1CB (targeted by 276 miRNAs), CALD1 (targeted by 170 miRNAs), RCC2 (targeted by 164 miRNAs) and HEXIM1 (targeted by 146 miRNAs). The interaction network between AS-LRGs and transcription factors (TFs) consisted of 10 genes and 212 transcription factors (Figure 6B). The top five predictions of the AS-LRG-TF interaction network encompassed: HEXIM1 (targeted by 176 TFs), PPP1CP (targeted by 28 TFs), CSRP1 (targeted by 21 TFs), CALD1 (targeted by 19 TFs) and WAS (targeted by 16 TFs). The AS-LRG–drug interaction network consisted of 11 drugs and 4 genes, as illustrated in Figure 6C.

### 3.4 Analysis of the relationship between immune infiltration and AS

**FIGURE 7:**
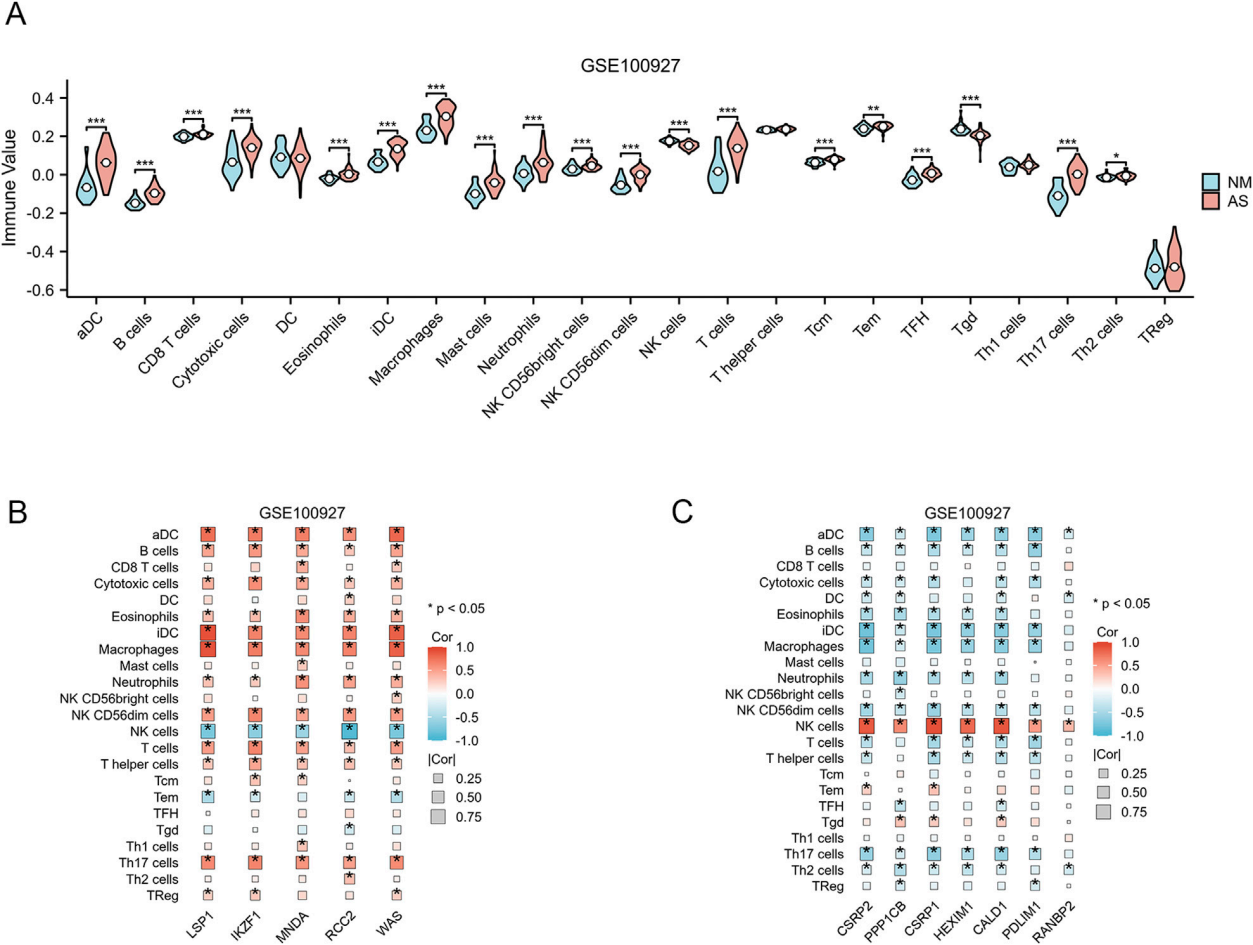
Analysis of Immune Infiltration. **(A)** Differences in the enrichment levels of 24 immune cell types within the GSE10092 dataset. (p < 0.05; *, p < 0.05; **, p < 0.01; ***, p < 0.001). **(B)** The association between 24 immune cell types and 5 upregulated AS-LRGs in the GSE100927 dataset. **(C)** The association between 24 immune cell types and 7 downregulated AS-LRGs in the GSE100927 dataset.

To achieve a more comprehensive understanding of the role of immune cells in AS and their association with critical genes, we employed the single-sample GSEA methodology to assess immune infiltration within the GSE100927 dataset. Among the 24 immune cell subpopulations, we observed significant differences between AS-affected tissues and normal tissues. The violin plot illustrated that AS patients exhibited varying levels of the majority of immune cell types (Figure 7A). Five upregulated genes and seven downregulated genes were identified as being associated with each immune cell type, as illustrated in Figures 7B, C. The chart indicates that the majority of upregulated genes exhibited a positive correlation with most immune cell types, with the exception of NK cells. Conversely, the chart shows that the majority of downregulated genes exhibited a negative correlation with most immune cell types, with the exception of NK cells.

### 3.5 Analysis of AS-LRGs in early and advanced lesions

**FIGURE 8:**
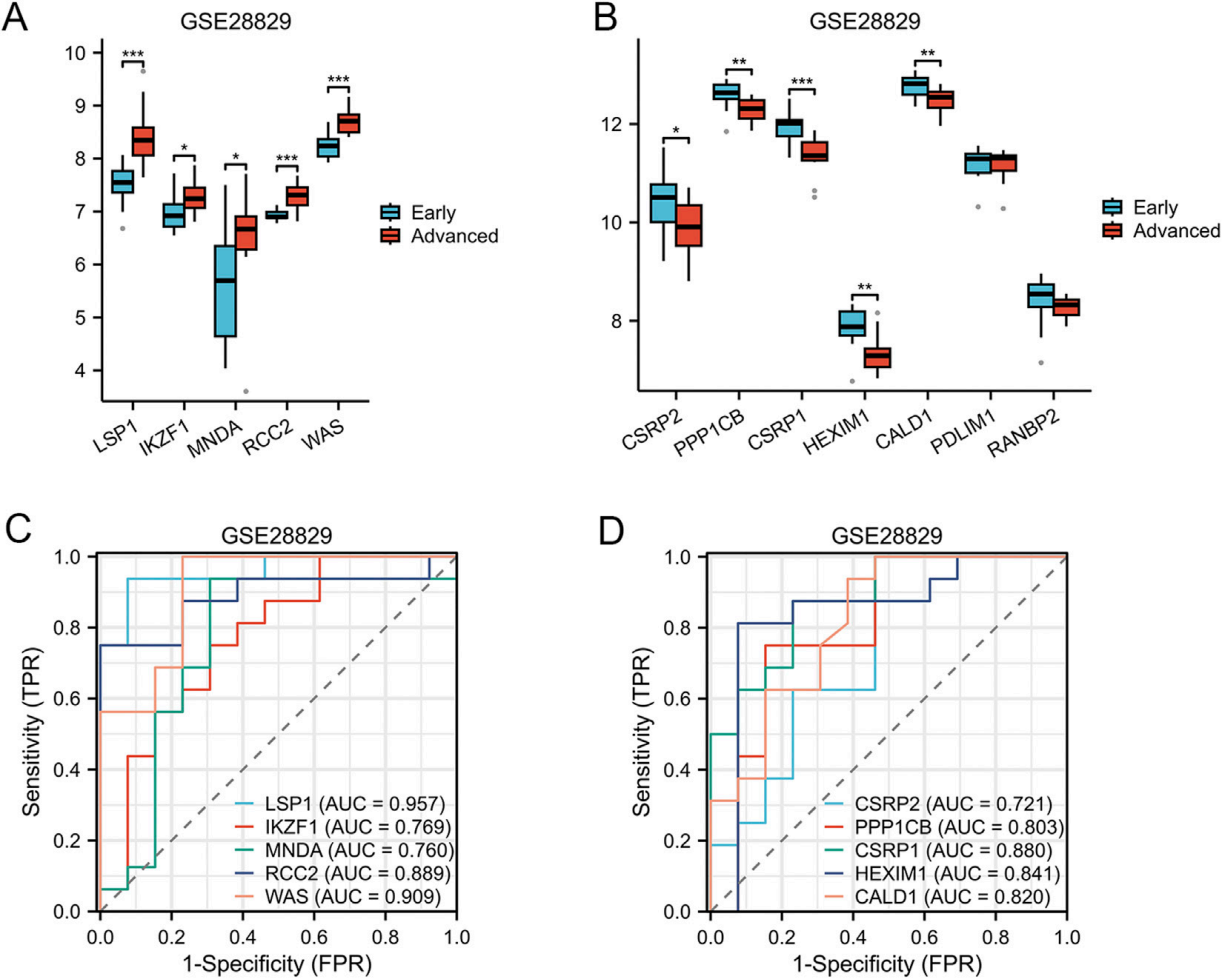
Expression Levels of AS-LRGs within the GSE28829 Dataset **(A, B)** The expression levels of the 5 upregulated AS-LRGs and 7 downregulated AS-LRGs in the GSE28829 dataset are depicted, with blue boxes representing early atherosclerotic plaques group and red boxes indicating advanced atherosclerotic plaques group. **(C, D)** Receiver operating characteristic (ROC) curve analysis for 5 upregulated AS-LRGs and 5 downregulated AS-LRGs in the GSE43292 dataset, with an area under the curve (AUC) greater than 0.7.

To evaluate the differential expression of AS-LRGs in early and advanced lesions, we utilized the GSE28829 dataset, which consists of 9 early lesion samples and 8 advanced lesion samples. The expression levels of the five upregulated genes and seven downregulated genes in the GSE28829 dataset were illustrated using box plots (Figures 8A, B). In the GSE28829 dataset, the five upregulated genes identified as having diagnostic value were LSP1, IKZF1, MNDA, RCC2, and WAS (AUC >0.7) (Figure 8C). The five downregulated genes identified as having diagnostic value were CSRP2, PPP1CB, CSRP1, HEXIM1, and CALD1 (AUC >0.7) (Figure 8D).

### 3.6 Analysis of AS-LRGs in the absence and presence of intraplaque hemorrhage

**FIGURE 9:**
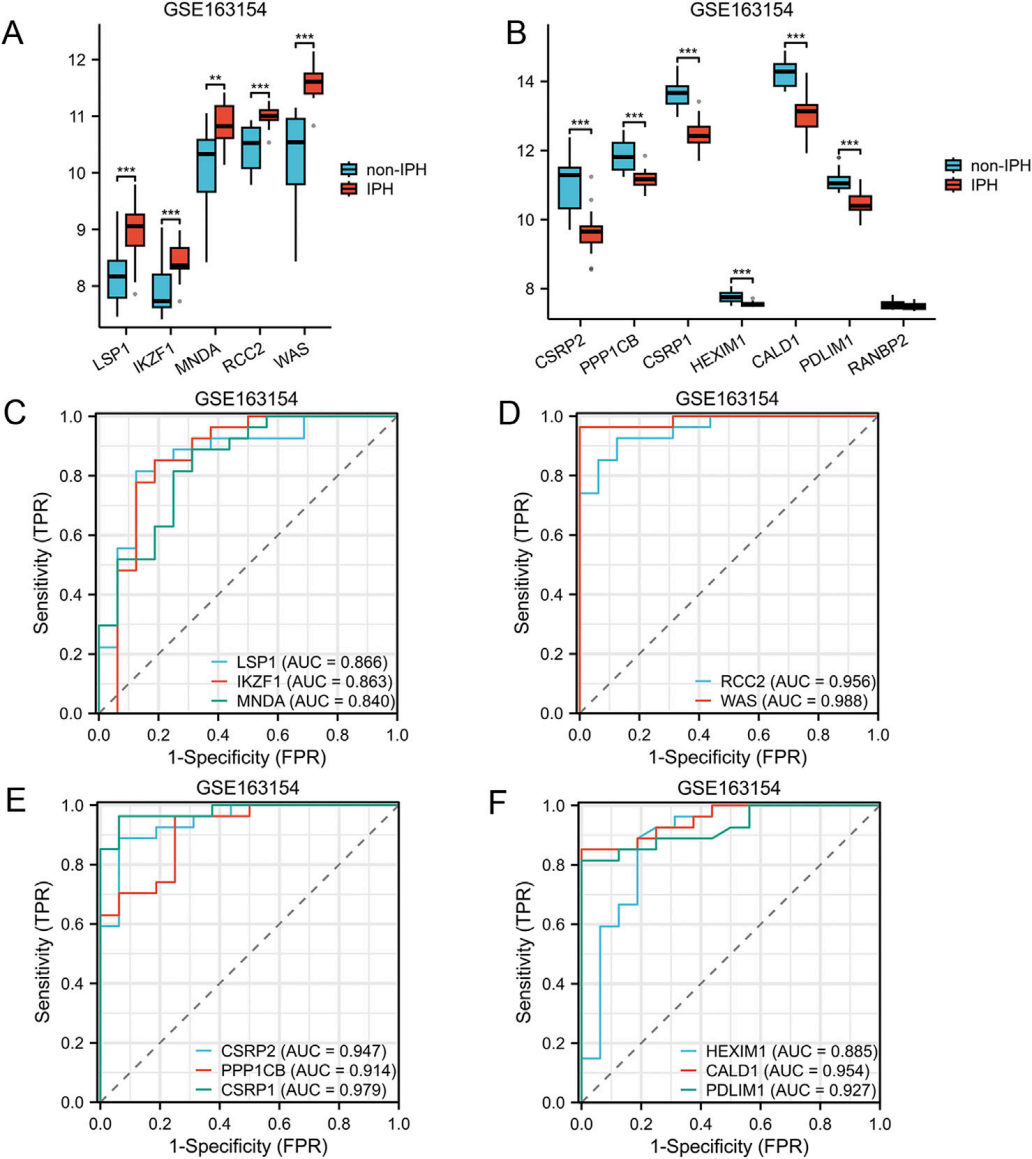
Expression Levels of AS-LRGs within the GSE163154 Dataset **(A, B)** The expression levels of the 5 upregulated AS-LRGs and 7 downregulated AS-LRGs in the GSE163154 dataset are illustrated, with blue boxes denoting the absence of intraplaque hemorrhage group and red boxes indicating the presence of intraplaque hemorrhage group. **(C–F)** Receiver operating characteristic (ROC) curve analysis for 5 upregulated AS-LRGs and 6 downregulated AS-LRGs in the GSE163154 dataset, with an area under the curve (AUC) exceeding 0.7.

The GSE163154 dataset, which includes samples indicating the presence or absence of intraplaque hemorrhage, was utilized to assess the relationship between AS-LRGs and intraplaque hemorrhage. The expression levels of the five upregulated genes and seven downregulated genes in the GSE163154 dataset were illustrated using box plots (Figures 9A, B). In the GSE163154 dataset, the five upregulated genes identified as having diagnostic value were LSP1, IKZF1, MNDA, RCC2, and WAS (AUC >0.7) (Figures 9C, D). The six downregulated genes identified as having diagnostic value were CSRP2, PPP1CB, CSRP1, HEXIM1, CALD1 and PDLIM1 (AUC >0.7) (Figures 9E, F).

## 4 Discussion

Atherosclerosis, characterized by the development of fibrofatty lesions within the arterial wall, significantly contributes to global morbidity and mortality from related diseases. It accounts for the majority of myocardial infarctions and numerous strokes and is a leading cause of disabling peripheral artery disease (Libby et al., 2019). Lactate, traditionally considered a mere byproduct of glycolysis, has garnered increasing recognition in recent years for its significant biological functions (Ouyang et al., 2023). Similarly, the significance of lactate in atherosclerosis has increasingly been acknowledged. Strategies aimed at targeting lactate production, regulating its transport, and modulating circulating levels may emerge as promising therapeutic approaches for atherosclerosis in the future (Li X. et al., 2024).

This study investigates the differential expression of LRGs in the context of AS. Previous research has highlighted the critical role of lactylation in cellular metabolism and signal transduction (Wang J. et al., 2022); however, the specific contributions of LRGs to the pathophysiology of AS remain poorly understood. We conducted a comprehensive analysis of the functional enrichment of these AS-LRGs, along with their associated transcription factors (TFs), microRNAs (miRNAs), and drugs, aiming to establish a solid foundation for subsequent in-depth investigations into their mechanisms. Moreover, the analysis of immune responses revealed that LRGs play a complex and multifaceted role in modulating immune cell activity within the atherosclerotic microenvironment. The findings demonstrated a significant positive correlation between several upregulated LRGs and immune cell infiltration, particularly in macrophages and T cells, highlighting their critical role in the inflammatory processes characteristic of atherosclerosis (Kuznetsova et al., 2020; Saigusa et al., 2020).

Atherosclerotic plaques typically develop over a period spanning several years to decades (Vergallo and Crea, 2020).However, thrombotic complications associated with atherosclerotic disease can arise abruptly and frequently without prior indication (Libby, 2013). The transition of atherosclerotic plaque from a stable to an unstable phenotype is associated with an increased incidence of acute cardiovascular events (Jin et al., 2021). Consequently, the assessment of plaque stability is of paramount importance. In this study, we investigated the expression differences of five upregulated genes and seven downregulated AS-LRGs in early versus advanced plaques, as well as high-risk versus low-risk plaques, along with their corresponding diagnostic ROC curves. We identified five upregulated genes and five downregulated AS-LRGs, both of which demonstrated significant differences and diagnostic efficacy between early and advanced plaques. Similarly, we identified five upregulated genes and six downregulated AS-LRGs, which also exhibited significant differences and diagnostic efficacy between high-risk and low-risk plaques. Understanding the fundamental mechanisms governing the transition from low-risk to high-risk rupture-prone plaques, as well as from early to advanced stages in human atherosclerosis, will facilitate early diagnosis and enable targeted interventions for cardiovascular diseases associated with atherosclerosis. Our forthcoming investigations will comprehensively assess the roles of these genes in the progression of atherosclerosis, along with their associated clinical significance.

The limitations of this study encompass the reliance on publicly available gene expression datasets lacking wet-lab validation, a relatively small sample size that may restrict the generalizability of the findings, and the potential for batch effects arising from the utilization of multiple datasets. These considerations underscore the imperative for future research to include larger cohorts and experimental validation in order to reinforce the clinical significance of the identified biomarkers.

In conclusion, this study conducted a systematic analysis of the expression patterns of lactylation-related genes in atherosclerosis, elucidating their potential roles in disease progression. The identification of these genes not only deepens our understanding of the molecular mechanisms underlying atherosclerosis but also establishes a foundation for future research aimed at developing innovative diagnostic and therapeutic strategies. Ultimately, the insights derived from this work may significantly enhance early diagnosis and personalized treatment options for patients with atherosclerosis.

## Data Availability

All data and analyses presented in this study are sourced from public databases, with all references clearly cited in the text.

## Nomenclature

AS: Atherosclerosis
LRGs: Lactylation-related genes
DEGs: Differentially expressed genes
AS-LRGs: Lactylation-related genes associate with atherosclerosis
GO: Gene Ontology
KEGG: Kyoto Encyclopedia of Genes and Genomes
ROC: Receiver Operating Characteristic
GSEA: Gene set enrichment analysis
ssGSEA: Single-sample GSEA
TFs: Transcription factors
